# Crowding by Invisible Flankers

**DOI:** 10.1371/journal.pone.0028814

**Published:** 2011-12-14

**Authors:** Cristy Ho, Sing-Hang Cheung

**Affiliations:** Department of Psychology, The University of Hong Kong, Hong Kong Special Administrative Region, China; University of California, Berkeley, United States of America

## Abstract

**Background:**

Human object recognition degrades sharply as the target object moves from central vision into peripheral vision. In particular, one's ability to recognize a peripheral target is severely impaired by the presence of flanking objects, a phenomenon known as visual crowding. Recent studies on how visual awareness of flanker existence influences crowding had shown mixed results. More importantly, it is not known whether conscious awareness of the existence of both the target and flankers are necessary for crowding to occur.

**Methodology/Principal Findings:**

Here we show that crowding persists even when people are completely unaware of the flankers, which are rendered invisible through the continuous flash suppression technique. Contrast threshold for identifying the orientation of a grating pattern was elevated in the flanked condition, even when the subjects reported that they were unaware of the perceptually suppressed flankers. Moreover, we find that orientation-specific adaptation is attenuated by flankers even when both the target and flankers are invisible.

**Conclusions:**

These findings complement the suggested correlation between crowding and visual awareness. What's more, our results demonstrate that conscious awareness and attention are not prerequisite for crowding.

## Introduction

Crowding is a breakdown in object perception whereby one's ability to recognize a peripheral target is severely impaired by the presence of flanking objects [1], [2]. Despite a significant number of studies on crowding since the work of Flom and colleagues in 1963 [3], [4], researchers have yet to agree on the mechanisms underlying the phenomenon of crowding and the cortical locus in visual processing at which crowding occurs (for a recent review, see [1], [2]). Attempts to pinpoint crowding to a specific stage in visual processing have been unsuccessful. Specifically, studies on early visual adaptation have reported substantially attenuated threshold-elevation aftereffect (TEAE) when the adapting grating was flanked (e.g., [5]). This has been taken to imply that crowding inaugurates at an early stage of cortical processing as the adaptation responsible for TEAE presumably occurs, at least in part, at V1 [6]. Parkes and colleagues [7] demonstrated mathematically that the compulsory averaging model might explain crowding (see also [8], [9]). Pelli [10], meanwhile, also showed mathematically that some properties of crowding might reflect the retinotopic and magnification properties of V1 and other visual areas. Tjan and Nandy [11] further proposed a computational model in an attempt to explain some properties of crowding by image statistics and lateral connections of V1 neurons.

Meanwhile, another line of studies have suggested that crowding originates beyond V1 (e.g., [12]–[14]). Bi and colleagues, for instance, reported fMRI evidence that orientation-selective adaptation in V2 and V3, but not in V1, was affected by crowding. Besides, Liu and colleagues [15] argued V4 to be the likely neural substrate of crowding. In particular, they capitalized on the dissociation between visual spatial distance and cortical distance and obtained results suggesting that the cortical locus of crowding was likely to be at a stage with contiguous hemifield representation. It is worth pointing out that their empirical findings suggested crowding at either V1 or V4. However, based on He et al's [14] findings, Liu and colleagues interpreted their findings as supporting evidence for V4 but not V1. It is yet interesting to note that one of the few neurophysiological studies on crowding found that V4 lesion caused little or no effect in the magnitude of crowding [16].

Few studies, to date, have investigated the role of conscious awareness in crowding. Chakravarthi and Cavanagh [17] attempted to determine the locus of crowding, considered to be a breakdown of feature integration, by manipulating the visibility of the flankers with three different kinds of masks: noise, metacontrast, and object substitution. While their masks were equally effective in masking the identity of the flankers, target recovery from crowding was observed only in the noise and metacontrast masking conditions but not with object substitution masking. Their results appear to suggest that crowding happens after V1 where noise masking sets in; at least it disrupts feature integration until after the early stages in the visual hierarchy when the low-level noise and metacontrast masks are effective in removing the flankers from visual awareness. Their study demonstrated that the effect of flanker visibility on crowding strength is contingent upon the specific method used to render the flankers invisible. Importantly, their study provided the only evidence in the literature that crowding persists even with invisible flankers that are removed from awareness by object substitution masking. Thus, the uncertain role of awareness in crowding seems to be attributable to the various different masking methods for controlling awareness.

In an attempt to investigate the relationship of flanker awareness and crowding in a tightly controlled manner, Wallis and Bex [18] designed a study in which the flankers were rendered perceptually invisible with the adaptation-induced blindness paradigm that allowed them to assess subjective awareness on a trial-by-trial basis. They found that crowding was not determined by the number of flankers that were physically present; instead it was correlated with the perceived number of flankers (i.e., visual awareness). Importantly, the researchers concluded that crowding is dependent on awareness such that interference from peripheral objects will not occur prior to their entry into conscious awareness.

Given the apparent inconclusive findings on the role of awareness in crowding in the limited literature, the present study was designed to assess whether conscious awareness of the flankers (and also the target) is necessary for crowding to occur. Dichoptic suppression by means of continuous flash suppression (CFS) was used to render the flankers and target stimuli invisible despite their physical presence in the retina. CFS is an established means to reliably suppress input from the non-dominant eye for an extended duration [19], [20]. Many studies have shown that the identity of objects masked by CFS could still exert an effect on certain behaviors (e.g., [21]–[23]). This means that CFS operates at a stage after object identification and presumably no earlier than the site of feature integration. The use of CFS will allow us to directly examine, for the first time, whether crowding operates on target and flankers that are both perceptually invisible using an adaptation paradigm.

To address the question whether crowding is dependent on flanker awareness and to test the faulty-integration hypothesis for crowding, we measured the contrast threshold for identifying grating patterns under condition in which the flankers were perceptually suppressed by the simultaneous presentation of CFS stimuli. Upon establishing the effectiveness of invisible flankers on crowding, we proceeded to determine whether crowding would persist in condition when both the target and flankers were rendered invisible by measuring TEAE. We hypothesized that crowding effect would still be observable even when people were unaware of the flanking and flanked stimuli. Such findings would support the faulty-integration hypothesis for crowding that it occurs due to erroneous compulsory integration of signals from the target and flankers [7], [24]. Alternatively, however, if crowding was completely released under perceptual suppression, this would suggest that the locus of crowding occurs after the site of visual awareness late in visual processing, and that it is possibly modulated by voluntary attention [14]. A third possibility would be a partial release from crowding under perceptual suppression. This would suggest that visual awareness and voluntary attention can modulate crowding strength, but are not prerequisite for crowding.

## Results

### Grating Orientation Discrimination Experiment

**Figure 1:**
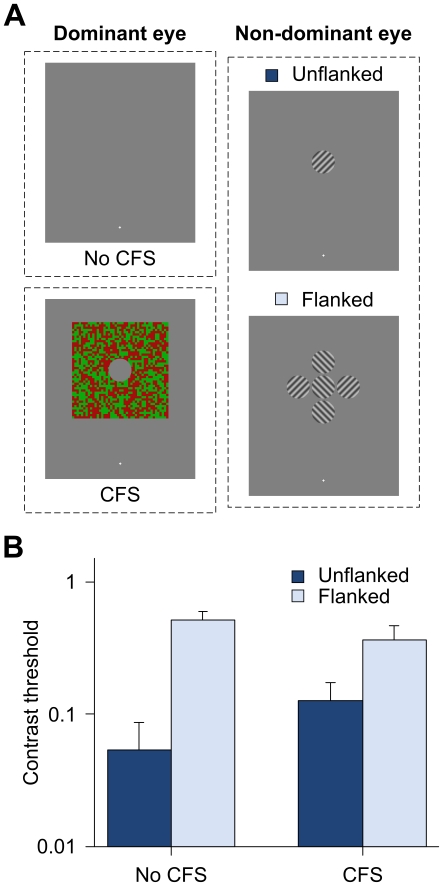
Results from the grating orientation discrimination experiment. (A) Stimuli presented in the experiment. In the CFS trials, flanking stimuli presented to the non-dominant eye of the observers in the flanked condition were perceptually suppressed from awareness by the CFS stimuli that were simultaneously presented to their dominant eye. Thus, in any given CFS trial, only the target grating pattern and the CFS stimuli were perceptually visible. Note that the actual contrast of the stimuli presented to their non-dominant eye was much lower than that illustrated here. (B) Contrast thresholds (in log scale) in the unflanked and flanked conditions as a function of CFS (n = 4). Error bars indicate the standard errors of the means.

In the first experiment, we assessed the effect of perceptually suppressed flankers on the contrast threshold for identifying the orientation of a target grating pattern (Figure 1A). Specifically, observers reported the orientation of the target grating (tilted left or right). Their awareness of the flankers was also assessed by having them indicate whether flankers were present or absent in each trial. The visibility of flankers presented to one eye (in the same eye to which targets were presented) was manipulated by the concurrent presentation of competing CFS stimuli in the other eye.

Linear mixed effects model was used to analyze the contrast threshold data. As shown in Figure 1B, an ANOVA revealed a significant interaction between the factors of Flanker and CFS [F(1, 41) = 14.44, p<0.001]. In particular, post-hoc pairwise comparisons showed significant crowding effects in both CFS conditions (both ps<0.001). In other words, despite the significant Flanker by CFS interaction, higher contrast thresholds were obtained for conditions when flankers were present than when the target was presented in isolation, no matter whether the flankers were suppressed or not. However, the magnitude of the crowding effect was stronger when the flankers were visible than when they were rendered invisible by the CFS stimuli. The main effect of Flanker was also significant [F(1, 41) = 113.51, p<0.001], indicating higher contrast thresholds for flankers present as compared to flankers absent trials, regardless of their visibility. The main effect of CFS was, however, not significant [F(1, 41) = 2.67, p = 0.110].

Forced-choice report on the visibility of flankers further asserted the effectiveness of our CFS manipulation. The observers correctly reported the presence (or absence) of flankers on 92.2% of trials (SEM = 3.1%) when no competing CFS stimuli were presented, with accuracy dropping to chance at 50.0% (SEM = 0.0%) in the CFS trials.

### Orientation-specific Adaptation Experiment

**Figure 2:**
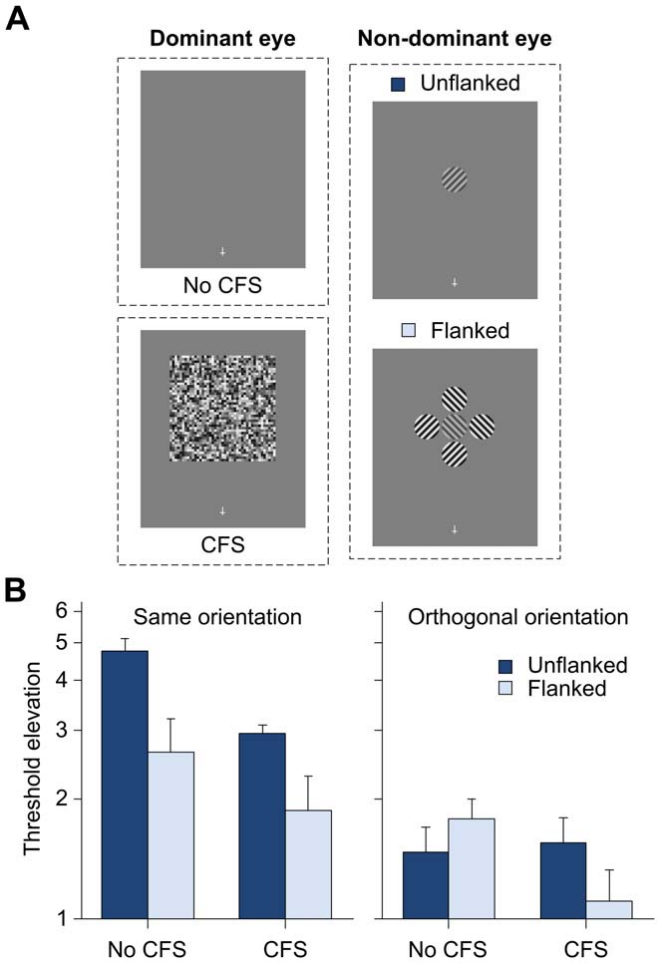
Results from the orientation-specific adaptation experiment. (A) Stimuli presented in the experiment. In the CFS trials, both the adaptor and flanking stimuli (if present) presented to the non-dominant eye of the observers were rendered perceptually invisible by the simultaneously presented CFS stimuli in their dominant eye throughout the adaptation phase. Thus, in any given CFS trial, only the CFS stimuli were perceptually visible during adaptation. Note that the actual contrast of the stimuli presented to the non-dominant eye was much lower than that illustrated here. (B) Strength of threshold-elevation aftereffect for test grating in the same orientation as the adapted grating (left panel) or orthogonal orientation relative to the adapted grating (right panel) in the unflanked and flanked conditions as a function of CFS (n = 4). Error bars indicate the standard errors of the means (in log scale).

In this experiment, we investigated further whether attenuated effect of flankers could still be observed when both the target and flankers were suppressed from awareness by measuring TEAE (Figure 2A). Specifically, upon prolonged exposure to target gratings (tilted left or right), contrast thresholds for test gratings (in the same or orthogonal orientation as the adapted gratings) were measured in two-interval-forced-choice (2IFC) detection task. A concurrent rapid serial visual presentation (RSVP) central task was also presented to ensure that observers maintained their fixation during the adaptation phase.

As shown in the left panel of Figure 2B, the analysis of threshold elevation data for the same orientation gratings with factors of Flanker and CFS revealed no interaction effect [F(1, 9) = 0.49, p = 0.549]. There were, however, significant main effects of Flanker [F(1, 9) = 22.43, p = 0.001] and CFS [F(1, 9) = 14.12, p = 0.005]. In particular, post-hoc pairwise comparisons showed the crowding effect for the same orientation gratings to be significant in both the No CFS (p<0.001) and CFS (p = 0.007) conditions, with a stronger crowding effect observed when CFS stimuli were not presented than in their presence.

A similar analysis of threshold elevation data after adapting to orthogonal target gratings revealed no interaction effect between the two factors [F(1, 9) = 3.81, p = 0.083]. In addition, both the main effects of Flanker [F(1, 9) = 0.25, p = 0.628] and CFS [F(1, 9) = 2.39, p = 0.16] were not significant. As shown in the right panel of Figure 2B, the TEAEs were relatively small (if present) and no effect of crowding was observed in either CFS conditions, consistent with previous findings on orientation-specific adaptation (e.g., [12]).

For the central fixation task, observers correctly counted the number of target crosses on 90.2% of trials (SEM = 0.9%) and 90.8% of trials (SEM = 2.2%) in the No CFS and CFS conditions, respectively. This suggests that our observers followed the instruction to fixate and performed the central task in a similar manner across different conditions during the experiment.

### Critical Spacing Experiment

One of the important criteria that define crowding relates to the critical spacing between the target and flankers [1], [25]. Essentially, the critical spacing of crowding operates as a function of target eccentricity, independent of target size. Bouma's rule further postulates the critical spacing at a given eccentricity to be approximately half the distance in eccentricity [26]. In this follow-up experiment, we investigated whether the critical spacing for crowding by invisible flankers would be consistent with Bouma's rule.

**Figure 3:**
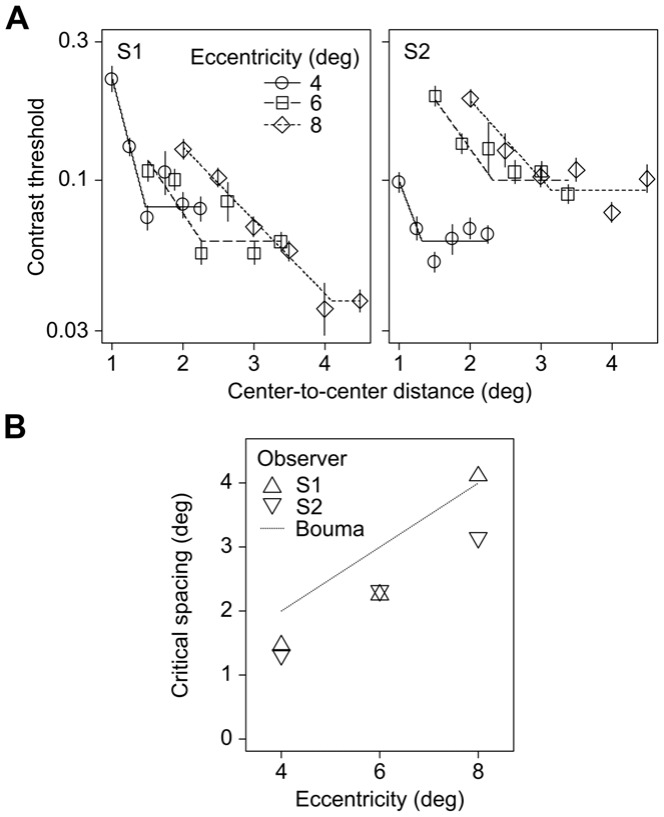
Results from the critical spacing experiment. (A) Individual observer data. Contrast thresholds (in log scale) for identifying target grating patterns presented in various degrees of eccentricity as a function of center-to-center distance between the target and flanking gratings. Error bars indicate the standard deviations (3 data points per condition was collected from each observer). Stimuli presented in this experiment were identical to those used previously in the CFS trials of the grating orientation discrimination experiment (Figure 1A). (B) The critical spacing of crowding (in degree) as a function of eccentricity (n = 2). Bouma's proportionality constant [23] is illustrated here for reference.

Critical spacing (*c*) at each eccentricity was estimated by fitting the following two-line function:
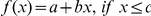


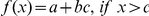
where *f(x)* was the log contrast threshold, *x* was the center-to-center distance, *a* was the y-intercept, *b* was the slope of the decreasing part of the function. With reference to Figure 3A, it can be seen that contrast thresholds for identifying a target grating decreased as the distance between the target and invisible flankers increased. In particular, the critical spacing at which the flankers ceased to have an effect on target identification set in at 1.47°, 2.26° and 4.11° (for S1), and 1.32°, 2.32°, and 3.15° (for S2) respectively for targets presented at 4°, 6°, and 8° eccentricity. This pattern of results was consistent with the widely reported findings that lie somewhere between 0.4–0.5 [25].

Forced-choice report on the visibility of flankers was again at chance level (33.3% as each block consisted of the unflanked condition and two flanked conditions with different center-to-center distances).

## Discussion

The present study was designed to investigate whether conscious awareness of the existence of the peripheral target and flankers is necessary in order for crowding to occur. Across different experiments, we manipulated the visibility of the target and flankers by suppressing them from perceptual awareness through the CFS technique. The results of the grating orientation discrimination experiment showed that the contrast thresholds required to identifying the orientation of a grating increased when the target was flanked, even when the flankers were rendered perceptually invisible. Our results therefore suggest that crowding represents a bottom-up information processing bottleneck. Such bottleneck persists even when people are unaware of the existence of the flanking objects that impair their ability to recognize objects in their peripheral visual field. The results of the orientation-specific adaption experiment further demonstrated the attenuation effect of invisible flankers. Parkes et al. [7] found that flanked local orientation signal of a target was averaged with that of the flankers. Consistent with the preattentive account of crowding [9], our results showed that adaptation of such flanked local orientation signal was being weakened relative to the unflanked adaptor even when the observers were completely unaware of the existence of both the target adaptor and flankers. Taken together, our results complement previous findings that flanker strength correlates with visual awareness [18] and demonstrate that flankers can act to impair feature integration prior to entering into conscious awareness.

Alternatively, one possible account for our observed pattern of results was lateral masking, that is, decreased sensitivity to target due to lateral inhibition by neighboring flankers at the sensory level early in visual processing [27], [28]. If our earlier results were indeed caused mainly by lateral masking rather crowding, one would expect to observe no proportionality constant between critical spacing and eccentricity [10]. Nevertheless, the results of the critical spacing experiment revealed evidence against such prediction. In particular, in line with Bouma's rule, crowding ceased to exist when the flankers were presented at locations beyond about 0.4 times the target eccentricity. These results therefore provide supportive evidence that our results indicate that crowding can occur even when the flankers are rendered invisible by CFS, and that a lateral masking account of our observation can be ruled out.

Previous studies of crowding in early visual adaptation were in favor of a voluntary attentional modulation account of crowding [12], [14], [29]. Covert attention has been shown to enhance spatial resolution via signal enhancement in the periphery where the original resolution may be too low for the task [30]. For instance, Bi and colleagues reported that orientation-specific TEAE was no longer affected by crowding once the attention of subjects were controlled for by having them perform a central luminance change detection task (cf. [5]). By contrast, the results of the present orientation-specific adaptation experiment advocate a dissociation of attention and awareness in the mechanism of crowding. Crowding exerted an effect on TEAE even when our observers were required to perform the central task having to count the number of red crosses that periodically showed up in amongst a rapidly-presented stream of distractor crosses. The attentional demand of our central task was presumably similar to that of the luminance change detection task in Bi et al.'s study. It should also be noted that attention is neither necessary nor sufficient for awareness [31], [32]. Given that orientation-specific adaptation occurs in the primary visual cortex [33], [34], our findings suggest that the site of crowding can occur at a relatively early stage in visual processing, apparently at or before the site of adaptation.

Other researchers, on the other hand, have argued that crowding happens beyond V1, or possibly in V4 or LOC, but definitely not in V1 (e.g., [1], [15]). In particular, Liu and colleagues provided evidence that crowding happens at a stage with contiguous hemifield representation, that is, either V1 or V4. Based on the results of other studies (e.g., [14]), they concluded that V4 was more likely to be the locus of crowding. The significant interaction between Flanker and CFS in our grating orientation discrimination experiment also seems to imply that crowding may happen at stages both before and after the locus of suppression by CFS. Some other studies have also reported crowding at high level (e.g., [35], [36]). When taken together, while these findings seem to suggest that crowding happens independently at multiple stages in visual processing [25], one may also argue that visual awareness and attention can modulate the strength of crowding at high level [37], with crowding itself being the result of faulty integration of features at relatively low level. It will be important for future studies to resolve this.

In addition, it should be noted that the present study provides one of the first pieces of evidence suggesting that crowding involves neuronal structures that do not correspond to, nor depend on, the neuronal correlates of conscious awareness. Other studies, to date, have reported an ‘all-or-none’ release of crowding when flankers are suppressed from visual awareness (e.g., [17], [18]). Consistent with Chakravarthi and Cavanagh's [17] object substitution findings, the first two experiments presented here showed a reduction of crowding in the CFS conditions, albeit not complete abolishment of crowding. Our findings therefore complement other studies that have demonstrated complete abolishment of crowding, with the discrepancy in ours and their findings being attributable to the extent of interference induced by the different paradigms chosen to suppress the flankers (e.g., adaptation-induced blindness in [18]; noise and metacontrast masks vs. object substitution mask in [17]). It is likely that the cortical locus of CFS occurs after the site of adaptation-induced blindness. In this respect, these results imply that awareness can modulate crowding strength even though it is not a prerequisite for crowding. Here, we argue that our manipulation of awareness by CFS provided clearly defined conscious and unconscious conditions for studying the role of awareness in crowding, the effectiveness of which was asserted by objective forced-choice report of awareness (cf. [22], [38]).

One may argue that the presentation of the central task in our orientation-specific adaptation experiment, if anything, complicates the design of our study given the proposal that attentional limits are the basis of crowding [14]. Since we have only presented a low attentional load task (cf. [39]), it would be interesting in future studies to examine what effects the presentation of putative high attentional load central task will have on crowding in the periphery. Specifically, it is important to investigate whether crowding occurs when the attentional limits of people are reached.

It is worth pointing out that although CFS and crowding are often used to manipulate visual awareness (e.g., [5]), the interaction between the two had not been addressed before. The results here imply that the two mechanisms operate separately. Crowding influences awareness by destroying the representation of object identity, while CFS does not. Thus, it is important for researchers to choose the technique appropriate for their research question concerned.

In summary, crowding represents faulty feature integration in peripheral vision. The experiments in the present study reliably demonstrated the findings of an attenuated effect of perceptually suppressed flankers on target identification. Taken together, these findings imply that crowding limits the spatial resolution of peripheral visual perception irrespective of conscious awareness and attention. The mechanism of crowding can occur at a relatively early stage in visual processing and it does not depend on visual awareness.

## Materials and Methods

Four young adult observers participated in each experiment, except for the critical spacing experiment in which two observers were tested. All observers had normal or corrected-to-normal visual acuity. The experiments were conducted in accordance with the guidelines laid down by the Human Research Ethics Committee for Non-Clinical Faculties, HKU. Written informed consent was obtained from all participants. Visual stimuli were displayed on a calibrated 17-inch CRT monitor set at a refresh rate of 85 Hz and 1024 by 768 pixels resolution. The background luminance was 17.8 cd/m^2^. Observers viewed the dicoptic display through angled mirrors in a darkened room from a distance of 40 cm, with the aid of a headrest and chinrest to stabilize fixation. In all experiments, target, flanker, and adaptor stimuli were always presented to the non-dominant eye of the observers, with the CFS stimuli being presented to their dominant eye.

### Grating Orientation Discrimination Experiment

In this experiment, target sinewave gratings of 2 cpd, 2.5° in diameter, randomly tilted at ±45°, were presented at an eccentricity of 10° in the upper visual field from the 0.25° fixation cross. In the flanked trials, four flanker sinewave gratings (two of which randomly oriented at 45° and the other two at −45°) of 15% Michelson contrast were presented around the target grating at center-to-center distance of 2.7° (Figure 1A). The CFS stimuli consisted of randomly generated red and green noise dots of size 0.25° that changed at 8.5 Hz. A 2.5° circular opening in the center of the CFS patches allowed visible perception of the target gratings (but not the flankers) when the patches were presented in the CFS condition. Contrast threshold was measured by two interleaved QUEST staircases (unflanked or flanked conditions) of 40-trial runs with a criterion of 82% and β of 3.5 [40], [41]. In each trial, the presentation of the 471 ms CFS stimuli (if present) led the 118 ms target by 353 ms. The observers had to report the orientation of the target grating (tilted left or right) and whether flankers were present in the trial (yes or no) by depressing the corresponding keys on the keyboard at the end of each trial. The order in which the observers performed the interleaved blocks of CFS trials (with or without) was counterbalanced across subjects. Log thresholds were averaged over three runs for each condition.

### Orientation-specific Adaptation Experiment

At the beginning of the experiment, the baseline contrast threshold (82% correct) for detecting the target grating in isolation was determined for each observer individually in a 2IFC task. The contrast of the adapting stimulus was then set at 4 times this baseline threshold, with the flanker contrast set at 8 times the baseline. The adapting stimulus consisted of a sinewave grating of 2 cpd, 2.5° in diameter that flickered in counterphase at 0.99 Hz to preclude afterimages. The orientation of the adaptor was randomly chosen to be either ±45°, which remained unchanged throughout each block. In each trial, an adaptor was presented at 10° eccentricity in the upper visual field for 5 s. This was followed by a 200 ms gap, after which a test grating of 153 ms was randomly presented in one of two successive intervals. The two intervals were delimited by 500 Hz beeps, separated by a gap of 400 ms. The test grating was either presented in the same or orthogonal orientation as the adaptor. In the flanked trials, four flanker sinewave gratings (two in the same and two in orthogonal orientation as the adaptor at random) were presented around the adaptor (at center-to-center distance of 2.7°) throughout the adaptation phase. In the CFS trials, patches of random grayscale noise dots (0.25° in size; changing at 8.5 Hz) were presented throughout the entire adaptation phase. Unlike the grating orientation discrimination experiment, the CFS patches had no opening and thus rendered both the adaptor and flankers perceptually invisible.

Contrast threshold for detecting the grating in the 2IFC task was measured by interleaved QUEST staircases (same or orthogonal test orientation relative to the adaptor) of 40-trial runs, with a criterion of 82% and β of 3.5. Threshold elevation was then calculated by dividing the contrast threshold for each condition by the baseline threshold. The order in which the observers performed the blocked Flanker (unflanked or flanked) by CFS (with or without) conditions was randomized across subjects.

A central attention-demanding RSVP task was also presented to ensure that the observers maintained fixation during the entire adaptation phase. The task was modeled after the low attention central task in van Boxtel et al.'s [39] study. Specifically, observers had to count the number of times (1, 2, 3, or 4) an upright or inverted red cross, presented in amongst crosses of different colors and randomized orientation, had appeared. Each cross (0.8° in height; 0.5° in width) in the RSVP stream was presented at fixation for 141 ms, followed by a blank of 141 ms, with two successive target crosses separated by at least one nontarget cross. At the end of each trial, the observers had to report the interval in which the test grating had appeared (first or second) and the number of red crosses that they had counted by depressing the corresponding keys on the keyboard.

### Critical Spacing Experiment

The design and procedure of the critical spacing experiment were identical to that of the grating orientation discrimination experiment, with the following exception. Target sinewave gratings presented were 4, 3, or 2 cpd; 0.75°, 1.125°, or 1.5° in diameter; for 4°, 6°, or 8° eccentricity, respectively. The flankers were fixed at 12.5% Michelson contrast and were presented at center-to-center distance of 0.25, 0.3125, 0.375, 0.4375, 0.5, or 0.5625 times the eccentricity. An opening in the center of the CFS patches with dot size of 0.125°, 0.1875°, or 0.25° respectively for 4°, 6°, 8° eccentricity allowed visible perception of the target gratings but not the flankers. In each block of trials, interleaved QUEST staircases (32-trial runs) of the unflanked condition and two flanked conditions with randomly chosen center-to-center distance, all in the same eccentricity, were presented. Log thresholds were averaged over three runs for each condition. Thus, in total, each observer performed 27 blocks of trials, in randomized order across subjects.
